# Narrative nursing to address negative emotions, body image disturbance, and satisfaction in patients after axillary odor surgery: a sequential explanatory mixed-methods study

**DOI:** 10.3389/fmed.2026.1745756

**Published:** 2026-03-25

**Authors:** Jianhua Tan, Jianfei Zhang, Kaixi Tan, Qiao Hu, Chuqing Wang

**Affiliations:** 1 Department of Burns and Plastic Surgery, Hengyang Medical School, The Second Affiliated Hospital, University of South China, Hengyang, China; 2 Department of Burns and Plastic Surgery, Hengyang Medical School, The affiliated Nanhua Hospital, University of South China, Hengyang, China; 3 Department of Plastic Surgery, Changsha Mylike Medical Cosmetic Hospital, Changsha, China

**Keywords:** axillary odor surgery, body image disturbance, narrative nursing, negative emotions, sequential explanatory mixed-methods study

## Abstract

**Background:**

Patients commonly experience negative emotions and body image disturbance following axillary odor surgery, while conventional nursing care often fails to adequately address their psychological needs. Narrative nursing, as a humanistic care model, offers a novel perspective for this issue.

**Methods:**

A sequential explanatory mixed-methods design was employed. In the quantitative phase, 120 post-operative axillary odor patients were randomly assigned to either an intervention group or a control group. Assessments were conducted using the SAS, SDS, BIRS and a satisfaction questionnaire. In the qualitative phase, 15 participants from the intervention group were purposively selected for semi-structured interviews, and the data were analyzed using Colaizzi’s phenomenological analysis method to extract themes.

**Results:**

Quantitative results showed that the intervention group had significantly lower scores on the SAS, SDS, BIRS, and significantly higher satisfaction scores compared to the control group (*p* < 0.001). Qualitative analysis yielded four themes: Emotional Catharsis and Psychological Liberation, Restructuring a Positive Illness Narrative and Self-Identity, Enhanced Sense of Control and Therapeutic Alliance, and Promotion of Mind-Body Integration and Quality of Life.

**Conclusion:**

Narrative nursing effectively alleviates negative emotions and body image disturbance in patients after axillary odor surgery and improves their satisfaction. The mechanism of action lies in helping patients externalize their problems and reconstruct a positive self-perception through the narrative process. It represents an effective model for transitioning from technical treatment to integrated mind-body care.

## 1 Background

Axillary odor, as a common physiological condition, poses a core challenge not rooted in organic pathology but rather in the severe psychological and social functional impairments it triggers. Epidemiological studies indicate this issue is particularly prevalent in East Asian populations. Individuals affected often encounter embarrassment and social rejection during interactions, leading to long-term struggles with anxiety, depression, and low self-esteem. At a deeper level, patients commonly experience significant body image disturbance—characterized by distorted perceptions of their own body and intense negative emotions—which profoundly impacts their self-identity and quality of life (1). Although surgical treatments, such as the minimally invasive technique focused on in this study (2), can effectively eliminate odor and provide physical relief, the surgery itself and the recovery process—including concerns about scarring and uncertainty regarding the final outcome—constitute an ongoing psychological stressor. Consequently, pre-existing negative emotions and body image disturbances may not dissipate immediately following the procedure (3).

However, current post-operative care models largely adhere to the traditional “disease-centered” paradigm, focusing intensely on monitoring physiological indicators and managing complications such as wound infections, hematomas, and pain (4). While this model is invaluable for ensuring patients’ physical safety, it systematically overlooks the identification and addressing of underlying psychological trauma. This fragmentation of “body-mind” care means that patients’ deep-seated emotional needs and requirements for cognitive restructuring lack professional support during the critical recovery period. Consequently, patients may find themselves in a predicament where “the body has healed, but the mind has not,” significantly hindering their comprehensive rehabilitation and quality of life.

To address this critical gap in clinical care, the concepts of narrative medicine and narrative nursing offer a robust theoretical perspective and practical toolkit (5). This approach posits that every patient is not merely a vessel of disease but also the holder and narrator of their unique illness story. It emphasizes that healthcare providers should engage in empathetic listening to deeply understand the impact of the illness on the patient’s life course (6). Core intervention techniques, such as “externalization” (separating the person from the problem), “deconstruction” (exploring the context behind the problem), and “reauthoring” (co-creating a new story), provide a structured pathway to help patients vent emotions and reshape a positive self-identity (7). Therefore, this study aims to apply the humanized intervention model of narrative nursing to the care of post-operative axillary odor patients. Utilizing a sequential explanatory mixed-methods approach, it seeks to comprehensively evaluate its efficacy in alleviating negative emotions, improving body image disturbance, and enhancing satisfaction, while also exploring its underlying mechanisms. The goal is to provide empirical evidence for promoting a paradigm shift from purely technical treatment to integrated mind-body care.

## 2 Materials and methods

### 2.1 Study design

This study employed a sequential explanatory mixed-methods design. This design was conducted in two consecutive phases: the first phase was a quantitative study utilizing a randomized controlled trial protocol, aiming to objectively evaluate the effect of the narrative nursing intervention on pre-defined outcome measures; the second phase was a qualitative study adopting a descriptive phenomenological approach, aiming to understand the lived experiences and subjective feelings of patients in the intervention group through in-depth interviews, thereby providing deep interpretation and contextual supplementation to the quantitative results from the first phase.

### 2.2 Participants

This study was conducted in the Department of Plastic Surgery outpatient clinic of the Second Affiliated Hospital of University of South China between January 2023 and January 2024. The inclusion criteria for participants were: (1) aged 18–45 years; (2) clinically diagnosed with primary axillary odor and undergoing a specified minimally invasive surgical procedure (2); (3) clear consciousness, possessing basic communication and reading comprehension abilities; (4) informed consent and voluntary participation in the study. Exclusion criteria were: (1) comorbid severe organic diseases (e.g., cardiac, hepatic, renal) or history of mental illness; (2) presence of cognitive or communication impairments preventing completion of interviews and scale assessments.

The sample size was calculated based on the primary outcome (Self-Rating Anxiety Scale, SAS score), referring to the effect size of previous similar narrative nursing intervention studies in surgical populations. We set a two-tailed α level of 0.05, a test power (1−β) of 0.9, an expected mean difference of 4.5 in SAS scores between the two groups, and a pooled standard deviation of 5.3. The calculation yielded a minimum sample size of 52 participants per group. Considering a potential 10% dropout rate during follow-up, we finally determined a sample size of 60 participants per group, with a total of 120 participants enrolled.

Based on statistical power analysis, the sample size was determined to be 120 participants. They were divided into an intervention group (*n* = 60) and a control group (*n* = 60) using a random number table. Furthermore, upon completion of the quantitative study, 15 participants from the intervention group who could provide rich information were selected via purposive sampling for qualitative interviews until data saturation was reached.

### 2.3 Intervention protocol

#### 2.3.1 Control group

Received routine postoperative care (8), which included: detailed wound care instructions (e.g., keeping the area dry, preventing infection), guidance on postoperative activity and dietary considerations, observation and management strategies for common complications (e.g., hematoma, subcutaneous effusion), and routine health education.

#### 2.3.2 Intervention group

In addition to routine care, received a standardized narrative nursing intervention delivered by uniformly trained and certified narrative nursing specialists (7). The intervention was completed in sessions within the first week post-surgery, following this core process:

#### 2.3.3 First session (postoperative day 1): establishing a safe relationship and externalizing the problem

The nurse established a trusting relationship through active listening and empathy. Using externalizing conversation techniques, the nurse guided the patient to describe “axillary odor” and associated issues like “embarrassment” and “inferiority” as objects separate from themselves. Example prompt: “If you were to give a name to this “embarrassment” that has troubled you for years, what would you call it? How has it step-by-step affected your social life?”

#### 2.3.4 Second session (postoperative days 2–3): deconstructing the story and identifying unique outcomes

The nurse deeply listened to the life story the patient had built around the “problem,” exploring the socio-cultural context and personal beliefs behind it. Simultaneously, the nurse identified “unique outcomes” acutely—moments where the patient demonstrated courage, strength, or exceptions to the problem-dominated story. Example prompt: “During times when “inferiority” almost controlled you, was there a moment where you successfully resisted it and acted like yourself?”

#### 2.3.5 Third session (postoperative days 5–7): re-authoring the new story and consolidating identity

The nurse and patient collaboratively reviewed the identified “unique outcomes,” linking them together to help the patient re-author a positive story centered on “resistance,” “growth,” and the “new self.” The new story and identity were ritualized and materialized through writing encouragement letters or creating therapeutic documents to consolidate the intervention effects.

### 2.4 Research tools and data collection

#### 2.4.1 Quantitative research tools

(1) General Information Questionnaire; 92) Self-Rating Anxiety Scale (SAS) (9) and Self-Rating Depression Scale (SDS) (10), with higher scores indicating more severe anxiety/depression; (3) Body Image Rating Scale (BIRS) (11), used to assess the degree of body image disturbance, with higher scores indicating greater disturbance; (4) Nursing Satisfaction Questionnaire [using a Likert five-point scale (12)].

#### 2.4.2 Data collection time points

Baseline data (SAS, SDS, BIRS) were collected from all patients before the intervention (T0, on the day of surgery). SAS, SDS, and BIRS scores were reassessed at 1 week post-intervention (T1). Satisfaction data were collected via telephone or outpatient review at 1-month post-intervention (T2).

#### 2.4.3 Qualitative data collection

After the T2 time point, one-on-one semi-structured interviews were conducted with the 15 purposively selected participants from the intervention group. The interview guide included questions such as: “What impressed you most about participating in this narrative nursing intervention?” and “How has this process influenced the way you view your surgical scars/past experiences?” Each interview lasted 30–45 min, was audio-recorded, and transcribed verbatim.

### 2.5 Data analysis

#### 2.5.1 Quantitative data analysis

Analyzed using SPSS 25.0 software. Categorical data were described using frequencies and percentages, and continuous data were described using mean ± standard deviation. Baseline comparisons between groups were performed using Chi-square tests or independent samples *t*-tests. Changes in SAS, SDS, and BIRS scores between the two groups across different time points (T0, T1) were compared using repeated measures ANOVA or generalized estimating equations. Satisfaction was compared using the Chi-square test. A *P*-value < 0.05 was considered statistically significant.

#### 2.5.2 Qualitative data analysis

Interview transcripts were analyzed using Colaizzi’s seven-step phenomenological analysis method (13). The steps included: familiarizing oneself with the transcripts, extracting significant statements, formulating meanings, clustering themes, developing a detailed description, producing the fundamental structure, and returning to participants for verification. Two researchers independently conducted the analysis and cross-checked to ensure the accuracy and credibility of the thematic extraction.

#### 2.5.3 Mixed-methods integration

In the results and discussion sections, joint displays and an explanatory sequential approach were used to directly connect the themes extracted from the qualitative study with the key findings from the quantitative study. Patients’ lived experiences and deep feelings were utilized to explain the underlying mechanisms of “why” and “how” the intervention worked, behind the quantitative data.

## 3 Results

### 3.1 Quantitative results

**FIGURE 1:**
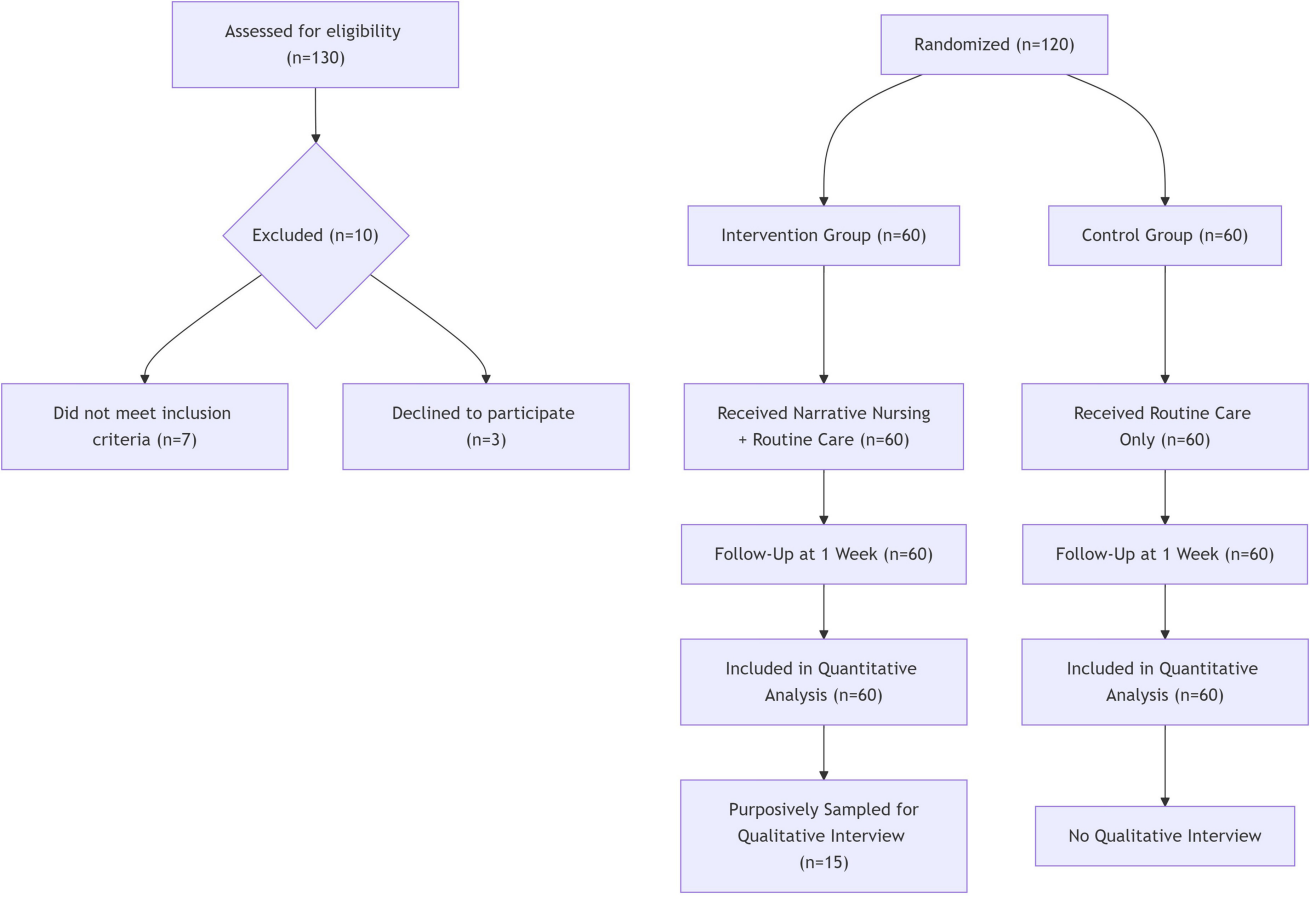
Participant flow diagram (CONSORT).

A total of 120 patients were enrolled in this study, with 60 each in the intervention and control groups. All patients completed the entire follow-up, and there were no dropouts. Details are shown in the CONSORT flow diagram (Figure 1). No statistically significant differences were found between the two groups in terms of age, gender, education level, or baseline SAS, SDS, and BIRS scores (*P* > 0.05), indicating comparability (Table 1).

**TABLE 1:** Comparison of baseline characteristics between the two groups.

Characteristic	Intervention group (*n* = 60)	Control group (*n* = 60)	Statistic	*P*-value
Age (years, mean ± SD)	25.8 ± 4.3	26.3 ± 4.8	*t* = −0.599	0.550
Gender [*n* (%)]				
Male	28 (46.67%)	25 (41.67%)	χ^2^ = 0.301	0.583
Female	32 (53.33%)	35 (58.33%)		
Education level [*n* (%)]				
≤High school	18 (30.00%)	15 (25.00%)	χ^2^ = 0.667	0.716
College	22 (36.67%)	25 (41.67%)		
≥Bachelor’s	20 (33.33%)	20 (33.33%)		
Disease duration (years, mean ± SD)	8.5 ± 3.1	8.2 ± 3.4	*t* = 0.502	0.616
Axillary odor severity [*n* (%)]				
Mild	12 (20.00%)	10 (16.67%)	χ^2^ = 0.454	0.797
Moderate	35 (58.33%)	38 (63.33%)		
Severe	13 (21.67%)	12 (20.00%)		
Baseline SAS score (mean ± SD)	55.18 ± 5.22	54.97 ± 5.41	*t* = 0.217	0.829
Baseline SDS score (mean ± SD)	56.89 ± 5.74	56.45 ± 5.88	*t* = 0.416	0.678
Baseline BIRS score (mean ± SD)	16.05 ± 3.28	15.94 ± 3.11	*t* = 0.190	0.850

Repeated measures ANOVA showed that at 1 week post-intervention (T1), the SAS, SDS, and BIRS scores of the intervention group were significantly lower than those of the control group, and the group-by-time interaction effect was statistically significant (*P* < 0.001). This indicates that the narrative nursing intervention was highly effective in alleviating negative emotions and reducing body image disturbance. Regarding nursing satisfaction, the survey results at 1-month post-intervention (T2) showed that the satisfaction rate in the intervention group was 96.67% (58/60), significantly higher than 81.67% (49/60) in the control group, and the difference was statistically significant (*P* < 0.01) (Table 2).

**TABLE 2:** Comparison of Self-Rating Anxiety Scale (SAS), Self-Rating Depression Scale (SDS), and Body Image Rating Scale (BIRS) scores between the two groups before and after intervention ($\bar{\text{x}}$ ± s).

Group	Time point	SAS score (mean ± SD)	SDS score (mean ± SD)	BIRS score (mean ± SD)
Intervention group (*n* = 60)	T0	55.18 ± 5.22	56.89 ± 5.74	16.05 ± 3.28
	T1	45.32 ± 4.17	46.78 ± 4.35	10.25 ± 2.36
Control group (*n* = 60)	T0	54.97 ± 5.41	56.45 ± 5.88	15.94 ± 3.11
	T1	52.46 ± 5.28	54.13 ± 5.62	15.87 ± 3.14

### 3.2 Qualitative results

Through Colaizzi’s phenomenological analysis of the interview data from 15 patients in the intervention group, four core themes were extracted:

#### 3.2.1 Theme 1: from emotional confinement to emotional catharsis

Most interviewees expressed that narrative nursing provided a safe and permitted “outlet” for them to release emotions accumulated over years. Patient P06 stated: “No one has ever talked with me so deeply about this matter; I always found it hard to talk about. After pouring out all the grievances and fears in my heart, it felt like a big stone was suddenly lifted from my chest.” This catharsis brought about a direct sense of psychological relief.

#### 3.2.2 Theme 2: restructuring the illness narrative and shifting self-identity

The intervention process helped patients reinterpret their relationship with axillary odor, shifting from seeing themselves as “defective individuals” to “survivors who have overcome difficulties.” Patient P12 shared: “I used to think of myself as a “person with a problem,” but after talking with the nurse, I realized I have been trying hard to get rid of it all along; I am actually brave. This surgery isn’t about covering up the problem, but a brave choice I made for myself.” This narrative restructuring fostered a positive self-identity.

#### 3.2.3 Theme 3: deepening of the therapeutic alliance and gaining a sense of control

Patients generally emphasized that the nurse’s deep listening and empathy during the narrative nursing intervention significantly enhanced their trust in the healthcare team and their own sense of control. Patient P03 expressed: “She (the nurse) truly understands me, not just completing a task. This makes me feel I’m not fighting alone, and I have more confidence in my recovery.”

#### 3.2.4 Theme 4: the rehabilitation journey: from physical healing to mind-body integration

Interviewees described how their rehabilitation goals expanded from simply “wound healing” to a “rebirth of their overall state.” Patient P09’s account was particularly typical: “Now when I look at the scar in the mirror, I don’t just think about whether it’s ugly or not, but I see it as a mark of me bidding farewell to the past and starting a new life. I feel that I have become much more relaxed and whole as a person.”

### 3.3 Mixed-methods integration

The results of the qualitative study provided explanations for the underlying mechanisms behind the significant findings of the quantitative study. Specifically:

1. The significant decrease in SAS and SDS scores in the intervention group aligned closely with the qualitative theme “From Emotional Confinement to Emotional Catharsis,” indicating that the improvement in scores stemmed from the substantial release and relief of patients’ internal emotions.
2. The significant improvement in BIRS scores in the intervention group can be profoundly explained by the qualitative theme “Restructuring the Illness Narrative and Shifting Self-Identity”: the shift in patients’ perception of their own body image (from negative to positive) was the internal psychological process underlying the reduction in body image disturbance.
3. The significant increase in satisfaction in the intervention group was closely related to the qualitative themes “Deepening of the Therapeutic Alliance and Gaining a Sense of Control” and “The Rehabilitation Journey: From Physical Healing to Mind-Body Integration.” This suggests that satisfaction stems not only from technical care but, more deeply, from the experience of being respected and understood through humanistic care and the achievement of a sense of holistic recovery.

## 4 Discussion

This study employed a sequential explanatory mixed-methods design to systematically investigate the effectiveness of narrative nursing in patients following axillary odor surgery. The quantitative results consistently demonstrated that, compared to the control group receiving routine care, the narrative nursing intervention significantly improved patients’ anxiety and depression, effectively reduced their body image disturbance, and substantially enhanced nursing satisfaction. Crucially, the subsequent qualitative study provided in-depth insights into the complex underlying mechanisms behind these quantitative findings, offering profound explanation and support for the scientific validity and effectiveness of this intervention model.

### 4.1 Interpretation of core findings: from quantitative data to qualitative experience

The four themes extracted from this study clearly outline the causal pathway and psychological processes through which narrative nursing produces positive outcomes.

First, the theme “From Emotional Confinement to Emotional Catharsis” directly explains the internal motivation behind the significant decrease in anxiety and depression scores (SAS, SDS). This finding aligns with Kleinman’s theory of “illness narratives,” which posits that when patients’ suffering is empathetically listened to and made meaningful, their emotional burden can be effectively alleviated (14). In this study, the shame, embarrassment, and anxiety that patients had accumulated over a long period due to axillary odor were able to be expressed and released in the safe and protective environment created by the nurses (15). This process itself possessed a powerful therapeutic effect, and it was the key factor that helped alleviate these negative emotions.

Second, the theme “Restructuring the Illness Narrative and Shifting Self-Identity” provides a key explanation for the significant improvement in body image disturbance (BIRS scores). Through the technique of “externalization,” narrative nursing successfully separated patients from the “axillary odor problem,” making them realize that “the problem is the problem, the person is the person.” Furthermore, through “deconstruction” and “reauthoring,” patients were guided to discover their own strengths and exceptional experiences in combating the problem, thereby reshaping an old narrative centered on “physical defect” and “social rejection” into a new narrative about “courage,” “autonomous decision-making,” and “growth.” This process profoundly corroborates the core concept of narrative therapy theory: changing the self-narrative that sustains the problematic issue is a prerequisite for change. When patients no longer view themselves as “individuals with odor” but as “survivors who have successfully overcome challenges,” their perception and evaluation of their body naturally become more positive.

Furthermore, the theme “Deepening of the Therapeutic Alliance and Gaining a Sense of Control” is closely related to the significant increase in satisfaction. Modern medicine increasingly emphasizes the importance of the doctor-patient partnership. The deep listening and co-creation advocated by narrative nursing transformed the traditional nurse-patient relationship from “technical guidance - passive acceptance” to a “humanistic empathy - collaborative coping” therapeutic alliance. This experience of being deeply understood and respected not only enhanced patients’ agency and sense of control during treatment but also directly constituted a core element of their high satisfaction with nursing care.

Finally, the synergistic effects of changes at the aforementioned three levels collectively contributed to the realization of the higher-order theme: “The Rehabilitation Journey: From Physical Healing to Mind-Body Integration.” This indicates that the ultimate benefit of narrative nursing transcends the mere alleviation of psychological symptoms; it promotes a paradigm shift in patients from focusing on “physical wound healing” to pursuing “holistic life quality,” which highly aligns with the comprehensive health perspective advocated by the biopsychosocial model.

### 4.2 Dialogue with existing literature and innovation

The findings of this study are consistent with research on narrative interventions in other fields, such as oncology and chronic pain, confirming their universal effectiveness in alleviating psychological distress (16, 17). However, the innovation and unique contribution of this study lie in its systematic application of narrative nursing to the post-operative axillary odor population—a group characterized by both physiological concerns and strong psychosocial attributes—and its pioneering use of mixed methods to empirically reveal the mechanism of action specifically through the unique pathway of “restructuring body image-related self-narrative” to improve body image disturbance. This not only validates the cross-problem applicability of narrative theory but also expands its practical boundaries in the field of body image disturbance intervention, providing new theoretical perspectives and empirical evidence for clinical work in this area.

### 4.3 Implications for clinical practice and dissemination

Based on the robust findings of this study, integrating narrative nursing into the standardized postoperative care pathways of plastic surgery, dermatology, and cosmetic medicine departments is clearly necessary and feasible. It is recommended that nursing managers proceed from the following aspects: First, implement systematic training and certification for core competencies in narrative nursing (e.g., externalizing conversations, deconstructive listening, reauthoring techniques) for specialist nurses. Second, develop a structured “Clinical Protocol Manual for Narrative Nursing” based on this study’s intervention protocol to ensure standardization and replicability of the intervention. Third, embed short, focused narrative dialogues at key time points in postoperative care (e.g., preoperative assessment, postoperative dressing changes, first follow-up visit) to efficiently institutionalize humanistic care and psychological support.

### 4.4 Limitations and future directions

Several non-modifiable design-related challenges in this study should be acknowledged, to clarify the potential biases and applicable boundaries of the research conclusions:

#### 4.4.1 Lack of assessor blinding and potential detection bias

Given the nature of this nursing intervention study, participants could not be blinded to group allocation, as they were fully aware of the nursing content they received. Meanwhile, we did not implement blinding for the outcome assessors responsible for scale data collection, which may introduce potential detection bias. It should be noted that the primary outcomes of this study (SAS, SDS, BIRS) were all patient self-reported scales, which reduced the subjective influence of assessors to a certain extent, but the risk of bias cannot be completely ruled out.

#### 4.4.2 Potential Hawthorne effect

The intervention group received three structured sessions of narrative nursing within the first postoperative week, resulting in longer face-to-face communication time with nurses compared with the control group that only received routine care. Therefore, we cannot fully exclude that the observed benefits may be partially derived from increased nursing attention and communication time, rather than the specific effects of the core techniques of narrative nursing.

#### 4.4.3 Single-center design and limited generalizability

This study was conducted in a single plastic surgery outpatient center, with all participants from the same regional population. Thus, the generalizability of the conclusions to other medical settings, regions, or ethnic populations needs further verification.

#### 4.4.4 Short-term follow-up and lack of long-term outcome data

The follow-up endpoint of this study was 1 month post-intervention, which only allowed us to observe the short-term effects of narrative nursing. The long-term sustainability of the intervention effects on patients’ body image, emotional state, and social function cannot be confirmed in this study.

Accordingly, future research could explore the following directions: conducting large-scale, multi-center randomized controlled trials to enhance the robustness and generalizability of the conclusions; implementing follow-ups over 6 months or 1 year to examine the long-term persistence of intervention effects; exploring narrative nursing models based on digital platforms (e.g., synchronous/asynchronous video consultation) to improve accessibility and efficiency; and evaluating the cost-effectiveness of this intervention model from a health economics perspective to inform decision-making regarding its dissemination at the healthcare policy level.

## 5 Conclusion

This study concludes that narrative nursing is an innovative intervention model that effectively alleviates negative emotions and body image disturbance in patients following axillary odor surgery, while significantly enhancing their satisfaction. Its mechanism of action is not merely simple psychological comfort, but rather operates by providing a safe channel for emotional catharsis, promoting a positive illness narrative and self-identity reconstruction, and deepening the therapeutic alliance, ultimately guiding patients toward comprehensive recovery through mind-body integration. Therefore, narrative nursing serves as a powerful tool for transitioning from the traditional biomedical model to the modern biopsychosocial medical model, and deserves promotion and application in clinical nursing practice.

## Data Availability

The original contributions presented in this study are included in this article/supplementary material, further inquiries can be directed to the corresponding author.

## References

[1] Zhang J Han P Yang F Jiang B. Advances in the treatment of axillary bromhidrosis. *Skin Res Technol.* (2024) 30:e13895. 10.1111/srt.13895 39096181 PMC11297419

[2] He Z Xi W Zhang J Han P Li X Yang F. Effect of optimizing the use of endoscope on the efficiency of microdynamic bromhidrosis removal operation. *J Cosmet Dermatol.* (2023) 22:2528–33. 10.1111/jocd.15753 36992574

[3] Huang Y Yang C Chen Y Chen C Lee S. Reduction in osmidrosis using a suction-assisted cartilage shaver improves the quality of life. *Dermatol Surg.* (2010) 36:1573–7. 10.1111/j.1524-4725.2010.01685.x 20722661

[4] Lin M Liu S. Health and health related outcomes of Chinese adult surgical patients managed by a nurse-led discharge service: a meta-analysis. *Int J Nurs Stud Adv.* (2025) 9:100434. 10.1016/j.ijnsa.2025.100434 41209814 PMC12590015

[5] Ga H. Shifting from a provider-centered to a person-centered model of long-term care for older patients in Korea: a narrative review. *Ewha Med J.* (2025) 48:e60. 10.12771/emj.2025.00794 41223889 PMC12611424

[6] Kalitzkus V Matthiessen P. Narrative-based medicine: potential, pitfalls, and practice. *Perm J.* (2009) 13:80–6. 10.7812/TPP/09.996 21373252 PMC3034473

[7] Wang G Pan S. Integrating narrative therapy and sleep intervention to enhance recovery, nutritional reconstitution, and 24-month survival following curative gastrectomy: a randomized controlled trial. *J Cancer Surviv.* (2025): 10.1007/s11764-025-01909-y Online ahead of print. 41225165

[8] Zhang J Han P Yang F Jiang B Tang Y Xiao X. Power-assisted rotary cutter with negative pressure suction through small incision for axillary osmidrosis. *Dermatol Ther.* (2022) 35:e15615. 10.1111/dth.15615 35656571

[9] Guo K Lu Z Deng H Shao Y. Risk factors of acne recurrence after treatment and establishment of an early warning model. *J Cosmet Dermatol.* (2025) 24:e70545. 10.1111/jocd.70545 41221639 PMC12606389

[10] Lin M Chen Y Zou Q Wang Z Chen Z Liu Y. Standardized pharmaceutical service improves medication adherence and reduces anxiety and depression in patients with chronic obstructive pulmonary disease. *Expert Rev Pharmacoecon Outcomes Res.* (2026) 26:107–14. 10.1080/14737167.2025.2586651 41218932

[11] Battistello C Remor E Costa ÍM de Oliveira ME Damin APS. Association between body image and quality of life of women who underwent breast cancer surgery. *Int J Environ Res Public Health.* (2025) 22:1114. 10.3390/ijerph22071114 40724182 PMC12294835

[12] Sweet L Elsbernd P Kiley J. The effect of a multi-force international educational experience on military physician Trainees’ perceptions of operational preparedness. *Mil Med.* (2025): 10.1093/milmed/usaf562 Online ahead of print. 41223051

[13] Çınarlı T Şener A Kıymaz D Saraçoğlu E Kılıç Ü. Nurses’ experiences responding to a devastating earthquake: the Kahramanmaraş case. *BMC Nurs.* (2025) 24:1378. 10.1186/s12912-025-04008-6 41214686 PMC12604363

[14] Soundy A Moffatt M Yip N Heneghan N Rushton A Falla D et al. Illness narrative master plots following musculoskeletal trauma and how they change over time, a secondary analysis of data. *Behav Sci.* (2024) 14:1112. 10.3390/bs14111112 39594412 PMC11591027

[15] Cepeda M Chapman C Miranda N Sanchez R Rodriguez C Restrepo A et al. Emotional disclosure through patient narrative may improve pain and well-being: results of a randomized controlled trial in patients with cancer pain. *J Pain Symptom Manage.* (2008) 35:623–31. 10.1016/j.jpainsymman.2007.08.011 18359604

[16] Wise M Marchand L Roberts L Chih M. Suffering in advanced cancer: a randomized control trial of a narrative intervention. *J Palliat Med.* (2018) 21:200–7. 10.1089/jpm.2017.0007 29135330 PMC5797325

[17] Lew Y Xin X. Using a narrative practice approach to understand in-depth experiences of individuals coping with chronic pain. *Pain Med.* (2021) 22:191–202. 10.1093/pm/pnaa223 32827046

